# Targeting adenosine monophosphate-activated protein kinase (AMPK) in preclinical models reveals a potential mechanism for the treatment of neuropathic pain

**DOI:** 10.1186/1744-8069-7-70

**Published:** 2011-09-21

**Authors:** Ohannes K Melemedjian, Marina N Asiedu, Dipti V Tillu, Raul Sanoja, Jin Yan, Arianna Lark, Arkady Khoutorsky, Jessica Johnson, Katherine A Peebles, Talya Lepow, Nahum Sonenberg, Gregory Dussor, Theodore J Price

**Affiliations:** 1 Department of Pharmacology, University of Arizona, N Campbell Ave, Tucson, 85724, USA; 2 Department of Biochemistry, McGill University, Sir William Osler, Montreal, H3G 1Y6, Canada; 3 Goodman Cancer Research Centre, McGill University, McGill University, Sir William Osler, Montreal, H3G 1Y6, Canada; 4 Graduate Interdisciplinary Program in Neuroscience, University of Arizona, N Campbell Ave, Tucson, 85724, USA; 5 Bio5 Institute, University of Arizona, N Campbell Ave, Tucson, 85724, USA

## Abstract

Neuropathic pain is a debilitating clinical condition with few efficacious treatments, warranting development of novel therapeutics. We hypothesized that dysregulated translation regulation pathways may underlie neuropathic pain. Peripheral nerve injury induced reorganization of translation machinery in the peripheral nervous system of rats and mice, including enhanced mTOR and ERK activity, increased phosphorylation of mTOR and ERK downstream targets, augmented eIF4F complex formation and enhanced nascent protein synthesis. The AMP activated protein kinase (AMPK) activators, metformin and A769662, inhibited translation regulation signaling pathways, eIF4F complex formation, nascent protein synthesis in injured nerves and sodium channel-dependent excitability of sensory neurons resulting in a resolution of neuropathic allodynia. Therefore, injury-induced dysregulation of translation control underlies pathology leading to neuropathic pain and reveals AMPK as a novel therapeutic target for the potential treatment of neuropathic pain.

## Background

Neuropathic pain is a debilitating condition wherein a large cohort of patients fail to achieve even partial pain relief [1]. Hence, novel treatment approaches targeting molecular mechanisms of pathology induced by peripheral nerve injury (PNI) are needed. PNI leads to changes in sensory neuron phenotype and function resulting in hyperexcitability and ectopic activity in these neurons driving neuropathic pain [2]. The important role of translation regulation in learning and memory has elucidated translation control as a critical factor for neuronal plasticity [3]. Multiple lines of evidence suggest that translation regulation at the level of the primary afferent neuron is crucial for the establishment and maintenance of enhanced pain states [4-9]. Several recent reports have suggested an important role for the mammalian target of rapamycin complex 1 (mTORC1) pathway in neuropathic pain [4,6]; however, mechanistic links between mTORC1 and pathology induced by PNI are still lacking. Moreover, treatment strategies that target translation control have not been clearly identified as potential treatments for neuropathic pain.

Translation control is orchestrated by upstream kinases that signal to the translation machinery [10]. These kinases can be targeted individually by selective inhibitors or they can be negatively modulated by endogenous signaling factors that act on these pathways [11]. A crucial kinase for negative regulation of translation is the ubiquitous, energy-sensing kinase AMP-activated protein kinase (AMPK). Activation of AMPK by depletion of cellular nutrients or through pharmacological intervention results in a dampening of signaling to the translation machinery [11] but the potential effects of AMPK activation on neuronal excitability, an important component of neuropathic pain conditions [2], is not known. AMPK can be targeted pharmacologically via a number of investigational compounds (e.g. AICAR and A769662) and by the widely clinically available and relatively safe drug metformin.

Herein we have tested the hypothesis that AMPK may represent a novel and efficacious opportunity for the treatment of chronic neuropathic pain. We find that PNI is linked to reorganization of translation machinery in injured nerves and demonstrate that pharmacological activation of AMPK leads to normalization of aberrant gene expression at the level of translation, decreased sensory neuron excitability and the resolution of neuropathic allodynia in preclinical models. Importantly, these effects are achieved by metformin suggesting an immediately available novel avenue for the potential treatment of neuropathic pain in humans. Hence, AMPK represents a novel therapeutic target for the treatment of neuropathic pain disorders.

## Results

### PNI induces a major reorganization of translational machinery in the peripheral nervous system

**Figure 1:**
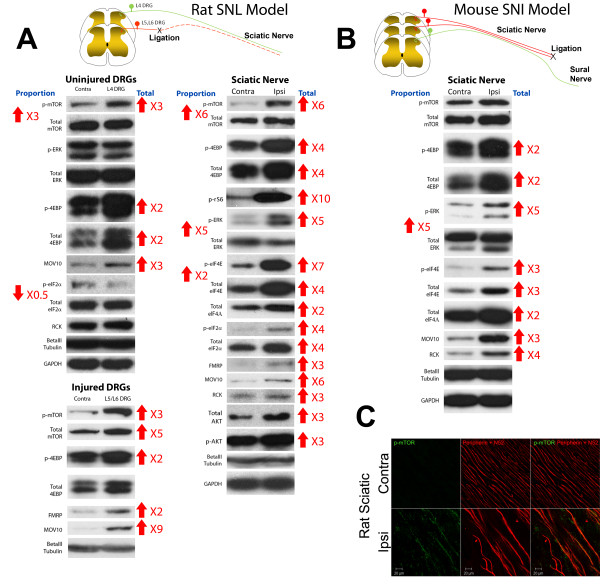
**PNI induces a fundamental reorganization of translation signaling and machinery in the injured PNS**. **A) **PNI in rats causes upregulation of translation machinery and signaling pathways associated with protein translation. These changes are observed in the injured sciatic nerve and in both injured (L5 and L6) and uninjured (L4) DRGs. **B) **PNI in mice increases proteins that enhance translation and associated signaling pathways. In both panels, up arrows indicate an increase while down arrows indicate a decrease; numbers indicate fold change in ipilateral vs. contralateral (right) and phospho-protein vs. total protein (left). **C) **Immunohistochemical colocalization of p-mTOR with sensory neuronal markers (peripherin + N52) show that activated mTOR localizes to axons of injured sciatic nerve from rats with SNL.

We utilized the rat spinal nerve ligation (SNL) and mouse spared nerve injury (SNI) models to assess biochemical changes in translation machinery occurring after PNI in the dorsal root ganglion (DRG) and sciatic nerve. SNL induced a significant increase in translation regulation signaling pathway components as well as translation machinery in the uninjured and injured DRG and in the sciatic nerve (Figure 1A). In the uninjured DRG (L4, Figure 1A) these changes included increased phosphorylation of mTOR, eukaryotic initiation factor (eIF) 4E binding protein (4EBP) and increased total 4EBP. Phosphorylation of eIF2α is negatively correlated with translation [12] and, consistent with enhanced translation in the L4 DRG, we observed decreased p-eIF2α (Figure 1A). Proteins associated with RNA processing and binding (e.g. Moloney leukemia virus 10 (Mov10) [13]) were also increased (see Table 1 for quantification). Likewise, in the injured DRGs (L5/6) p-mTOR and p-4EPB were increased as well as total mTOR. Increases in total Mov10 and fragile × mental retardation protein (FMRP) were also observed (Figure 1A, see Table 2 for quantification).

**Table 1:** Quantification of western blots on ipsilateral vs contralateral L4 (uninjured) DRGs from SNL rats 17 days post-surgery.

RAT SNL					
**Uninjured L4 DRG vs. Contralateral**					

	Contra	Contra	Ipsi	Ipsi	

Protein/Standardization	Mean %	SEM	Mean %	SEM	P-value

p-mTOR/mTOR	100	17.9	402.8	63.6	0.001

p-mTOR/beta III tubulin	100	19.0	425.7	92.0	0.012

mTOR/beta III tubulin	100	12.1	86.0	34.4	NS

p-4EBP/4EBP	100	11.6	100.1	9.3	NS

p-4EBP/beta III tubulin	100	15.8	190.3	22.2	0.003

4EBP/beta III tubulin	100	17.8	182.6	14.8	0.002

p-ERK/ERK	100	11.0	121.0	17.1	NS

p-ERK/beta III tubulin	100	19.8	111.3	13.2	NS

ERK/beta III tubulin	100	12.3	97.2	4.0	NS

p-eIF4alpha/eIF4alpha	100	13.5	60.1	8.0	0.026

p-eIF2alpha/beta III tubulin	100	19.7	61.8	7.2	NS

eIF2alpha/beta III tubulin	100	13.6	106.9	6.3	NS

mov10/beta III tubulin	100	20.8	220.9	37.0	0.008

rck/beta III tubulin	100	12.5	123.5	14.1	NS

eIF4A/beta III tubulin	100	15.3	102.5	14.7	NS

fbxw7/beta III tubulin	100	45.1	102.3	30.5	NS

p-AKT/total AKT	100	10.9	112.8	14.7	NS

p-AKT/beta III tubulin	100	6.0	107.8	12.2	NS

AKT/beta III tubulin	100	10.8	92.5	3.9	NS

p-mTOR/GAPDH	100	14.1	387.2	72.4	0.006

p-4EBP/GAPDH	100	13.3	162.6	12.0	0.002

4EBP/GAPDH	100	11.8	163.1	9.6	0.000

mov10/GAPDH	100	19.4	195.6	34.6	0.025

**Table 2:** Quantification of western blots on ipsilateral vs. contralateral L5 and L6 (injured) DRGs from SNL rats 17 days post-surgery.

RAT SNL					
**Injured L5/L6 DRG vs. Contralateral**					

	Contra	Contra	Ipsi	Ipsi	

Protein/Standardization	Mean %	SEM	Mean %	SEM	P-value

p-mTOR/mTOR	100.0	22.2	71.6	16.2	NS

p-mTOR/beta III tubulin	100.0	27.5	321.0	59.1	0.003

mTOR/beta III tubulin	100.0	23.6	579.4	178.3	0.016

p-4EBP/4EBP	100.0	12.7	98.4	7.3	NS

p-4EBP/beta III tubulin	100.0	33.1	222.1	46.2	0.049

4EBP/beta III tubulin	100.0	42.2	179.8	18.7	NS

FMRP/beta III tubulin	100.0	29.3	199.0	36.6	0.047

mov10/beta III tubulin	100.0	36.4	875.0	304.2	0.035

SNL induces a degeneration of axons from the L5 and L6 DRGs resulting in pathology to uninjured sciatic axons originating from L4 DRG [2] that comingle in the nerve. In the sciatic nerve, PNI led to enhanced mTOR and extracellular signalregulated kinase (ERK) pathway activity reflected by increased p-4EBP, p-ribosomal S6 protein (rS6p) and p-eIF4E, respectively. Upstream activation of mTOR in this setting is likely linked to enhanced AKT activity as increased p-AKT and p-mTOR at S2448 was observed [14]. We have recently shown that nerve growth factor (NGF) and interleukin 6 (IL-6) signal to the translational machinery in dorsal root ganglion (DRG) and trigeminal ganglion (TG) neuronal axons via mTOR and ERK, respectively [7]. We found that NGF (ipsilateral 1532 ± 185.6 pg/ml vs. contralateral 924.5 ± 55.7 pg/ml, p = 0.011) and IL-6 (ipsilateral 1282 ± 100.8 pg/ml vs. contralateral 988.6 ± 35.2 pg/ml; p = 0.021) were increased by PNI in the sciatic nerve suggesting that NGF and IL-6 may contribute to local mTOR and ERK activation following injury. We further noted increases in translational machinery (total eIF4E, eIF4A, eIF2α) and RNA processing and binding proteins (FMRP, Mov10 and rck/p54; Figure 1A, see Table 3 for quantification). Hence, these results demonstrate a fundamental reorganization of translation signaling pathways and machinery in the sciatic nerve induced by PNI.

**Table 3:** Quantification of western blots on ipsilateral vs. contralateral sciatic nerves from SNL rats 17 days post-surgery.

RAT SNL					
**Sciatic Nerve Ipsilateral vs. Contralateral**					

	Contra	Contra	Ipsi	Ipsi	

Protein/Standardization	Mean %	SEM	Mean %	SEM	P-value

p-mTOR/mTOR	100	27.6	581.8	112.0	0.002

p-4EBP/4EBP	100	8.9	117.0	13.6	NS

p-4EBP/4EBP	100	12.6	408.0	104.2	0.008

p-rS6/beta III-tubulin	100	22.6	1005.5	300.0	0.007

p-AKT/AKT	100	17.3	101.6	22.0	NS

p-AKT/beta III-tubulin	100	14.4	276.4	67.5	0.034

AKT/beta III-tubulin	100	11.2	261.6	36.9	0.003

p-ERK/ERK	100	8.8	510.3	153.4	0.023

p-eIF4E/eIF4E	100	14.8	164.0	17.6	0.024

p-eIF4E/beta III-tubulin	100	25.1	700.8	168.5	0.008

eIF4E/beta III-tubulin	100	16.8	415.4	62.7	0.001

eIF4A/beta III-tubulin	100	14.6	241.0	31.0	0.001

p-eIF2alpha/eIF2alpha	100	8.9	110.7	21.4	NS

p-eIF2alpha/beta III-tubulin	100	15.3	506.4	163.4	0.024

eIF2alpha/beta III-tubulin	100	19.5	575.7	107.9	0.001

FMRP/beta III-tubulin	100	14.2	257.8	59.0	0.024

mov10/beta III-tubulin	100	28.5	636.3	161.2	0.008

rck/beta III-tubulin	100	22.9	483.4	141.4	0.015

fbxw7/beta III-tubulin	100	37.7	305.7	72.9	0.028

p-4EBP/GAPDH	100	25.4	224.6	18.6	0.004

4EBP/GAPDH	100	26.1	257.4	32.5	0.006

p-eIF4E/GAPDH	100	15.9	458.0	54.4	0.000

eIF4E/GAPDH	100	10.6	270.2	12.4	0.000

p-eIF2alpha/GAPDH	100	10.3	297.4	74.9	0.018

eIF2alpha/GAPDH	100	4.7	244.3	15.7	0.000

rck/GAPDH	100	20.5	280.0	62.2	0.013

p-AKT/GAPDH	100	8.4	182.8	26.1	0.017

AKT/GAPDH	100	14.1	177.2	18.6	0.011

eIF4A/GAPDH	100	9.3	249.6	33.0	0.001

fbxw7/GAPDH	100	25.4	420.6	134.1	0.037

To verify that these findings are generalized to other models of PNI in other species, we utilized the mouse SNI model to confirm reorganization of biochemical components mediating translation regulation. In the injured sciatic nerve of the mouse SNI model we observed an upregulation of total 4EBP, eIF4E, eIF4A, Mov10 and rck/p54 and increased ERK activity (Figure 1B, see Table 4 for quantification). To demonstrate localization of enhanced mTOR signaling to DRG axons we used immunohistochemistry (IHC) for p-mTOR. Uninjured sciatic nerves from rats did not exhibit detectable levels of p-mTOR on serine 2448 (Figure 1C). However, following PNI robust p-mTOR IHC co-localized with markers of DRG neuron axons (peripherin + N52, Figure 1C). Collectively, these findings support the conclusion that PNI induces a fundamental reorganization of translation regulation signaling in the injured PNS and IHC findings with p-mTOR suggest that these changes occur in sensory neuron axons.

**Table 4:** Quantification of western blots on ipsilateral vs. contralateral sciatic nerves from SNI mice 17 days post-surgery.

MOUSE SNI					
**Sciatic Nerve Ipsilateral vs. Contralateral**					

	Contra	Contra	Ipsi	Ipsi	

Protein/Standardization	Mean %	SEM	Mean %	SEM	P-value

p-mTOR/mTOR	100	7.6	128.0	19.6	NS

p-mTOR/beta III tubulin	100	13.4	211.4	68.9	NS

mTOR/beta III tubulin	100	14.0	155.7	42.1	NS

p-4EBP/4EBP	100	5.8	94.0	5.7	NS

p-4EBP/beta III tubulin	100	15.0	150.6	17.6	0.049

4EBP/beta III tubulin	100	14.6	169.6	23.0	0.025

p-ERK/ERK	100	13.6	511.9	187.6	0.049

p-ERK/beta III tubulin	100	13.3	500.4	109.5	0.003

ERK/beta III tubulin	100	8.3	111.1	8.8	NS

p-eIF4E/eIF4E	100	25.5	104.9	13.1	NS

p-eIF4E/beta III tubulin	100	22.7	301.0	41.3	0.001

eIF4E/beta III tubulin	100	13.2	283.0	42.5	0.001

eIF4A/beta III tubulin	100	16.8	185.9	28.0	0.022

mov 10/beta III tubulin	100	31.6	256.9	41.0	0.011

rck/beta III tubulin	100	26.3	482.7	155.0	0.032

fbxw7/beta III tubulin	100	35.7	133.4	42.2	NS

### PNI enhances eIF4F complex formation and causes an increase in nascent protein synthesis in the sciatic nerve

**Figure 2:**
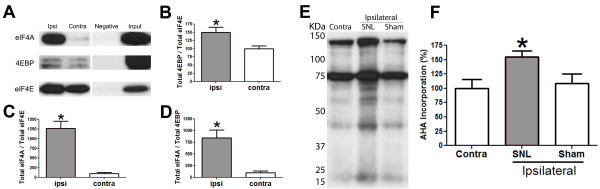
**PNI enhances cap-dependent protein translation in the injured sciatic nerve**. **A) **Western blot of eIF4A, 4EBP and eIF4E from sciatic nerve co-precipitated using 7-methyl-GTP conjugated beads. **B) **PNI induces only 50% increase in 4EBP (negative regulator of translation) association with cap-binding protein eIF4E, **C) **while injury induces a 1200% increase in the association of eIF4A (a component of eIF4F complex) with eIF4E. **D) **PNI induces a 750% increase in the ratio of eIF4A to 4EBP associated with eIF4E bound to 7-methyl-GTP conjugated beads. PNI increases nascent protein synthesis in injured sciatic nerve. **E) **Western blot of AHA incorporated into nascently synthesized proteins. **F) **PNI induces a 50% increase in the incorporation of AHA into nascently synthesized proteins. All samples taken 17 days post SNL from rats with n = 6 per condition. *p < 0.05.

To directly demonstrate stabilization of the eIF4F complex on the 5'-mRNA-cap we incubated proteins extracted from sciatic nerves ipsilateral and contralateral to SNL with sepharose beads conjugated to 7-methyl-GTP. Ipsilateral to the injury we observed 1200% increase in eIF4A association with the 7-methyl-GTP conjugated beads (Figure 2A and 2C). Conversely, only a modest increase in 4EBP was detected associated with 7-methyl-GTP (Figure 2A and 2B) despite a robust increase in total 4EBP induced by PNI (Figure 1A and 1B). The ratio of eIF4A to 4EBP (750% increase; Figure 2D) associated with 7-methyl-GTP strongly suggests a robust increase in cap-dependent translation in the sciatic nerve induced by PNI. To directly ascertain altered nascent protein synthesis following PNI we incubated excised sciatic nerves ipsilateral and contralateral to SNL with a click-chemistry compatible methionine analogue L-azidohomoalanine (AHA) [15,16]. AHA incorporation into nascently synthesized proteins was biotinylated using click-chemistry and detected by Western blotting. With 2 hr incubation, injured nerves incorporated 50% more AHA than either contralateral uninjured nerves or sciatic nerves taken from sham rats (Figure 2E and 2F). Taken together, these findings directly demonstrate that PNI-induced reorganization of translation regulation in the sciatic nerve results in enhanced eIF4F complex formation and increased protein synthesis.

### AMPK activators reverse PNI induced allodynia

**Figure 3:**
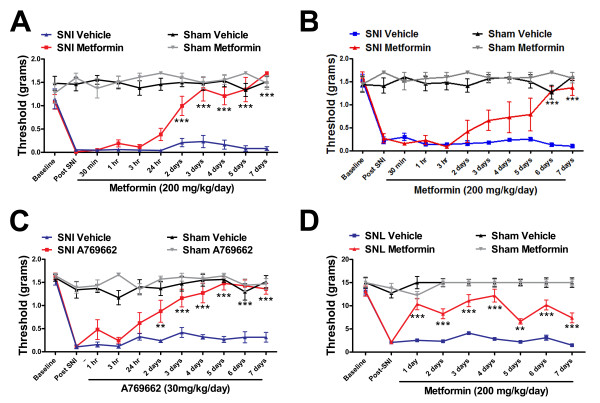
**AMPK activators reduce PNI-induced neuropathic allodynia**. Daily intraperitoneal injections of metformin in mice starting 2 **(A) **or 7 **(B) **weeks post PNI leads to a reversal of mechanical allodynia. **C) **A769662 daily treatment also reversed PNI-induced allodynia when started 2 weeks post PNI. **D) **Treatment of SNL rats (2 weeks post surgery) with metformin also significantly reduces neuropathic allodynia. N = 6 per group. **p < 0.01 and ***p < 0.001.

We hypothesized that activation of AMPK signaling may represent a novel mechanism for treatment of neuropathic pain. AMPK activation inhibits the mTOR pathway, is associated with decreased ERK activity [11] and inhibits insulin receptor substrate (IRS)-mediated feedback signaling [17]. Metformin stimulates the AMPK pathway through multiple mechanisms [18,19]. Mice treated with metformin (200 mg/kg/day [20] for 7 days) starting 2 (Figure 3A) or 7 (Figure 3B) weeks post-SNI displayed a complete reversal of tactile allodynia. No changes in threshold were observed in sham mice (Figure 3A and 3B). Likewise, treatment with A769662 (30 mg/kg/day [21] for 7 days), a positive allosteric modulator of AMPK [21], led to a full reversal of tactile allodynia with no effect in shams (Figure 3C). Metformin (200 mg/kg/day for 7 days) also alleviated SNL-induced neuropathic allodynia without influencing thresholds in sham rats (Figure 3D). No adverse motor effects were observed in sham or PNI animals. These results establish AMPK activators as a potentially efficacious class of drugs for the treatment of PNI-induced neuropathic pain.

### AMPK activators reverse PNI-induced biochemical changes

**Figure 4:**
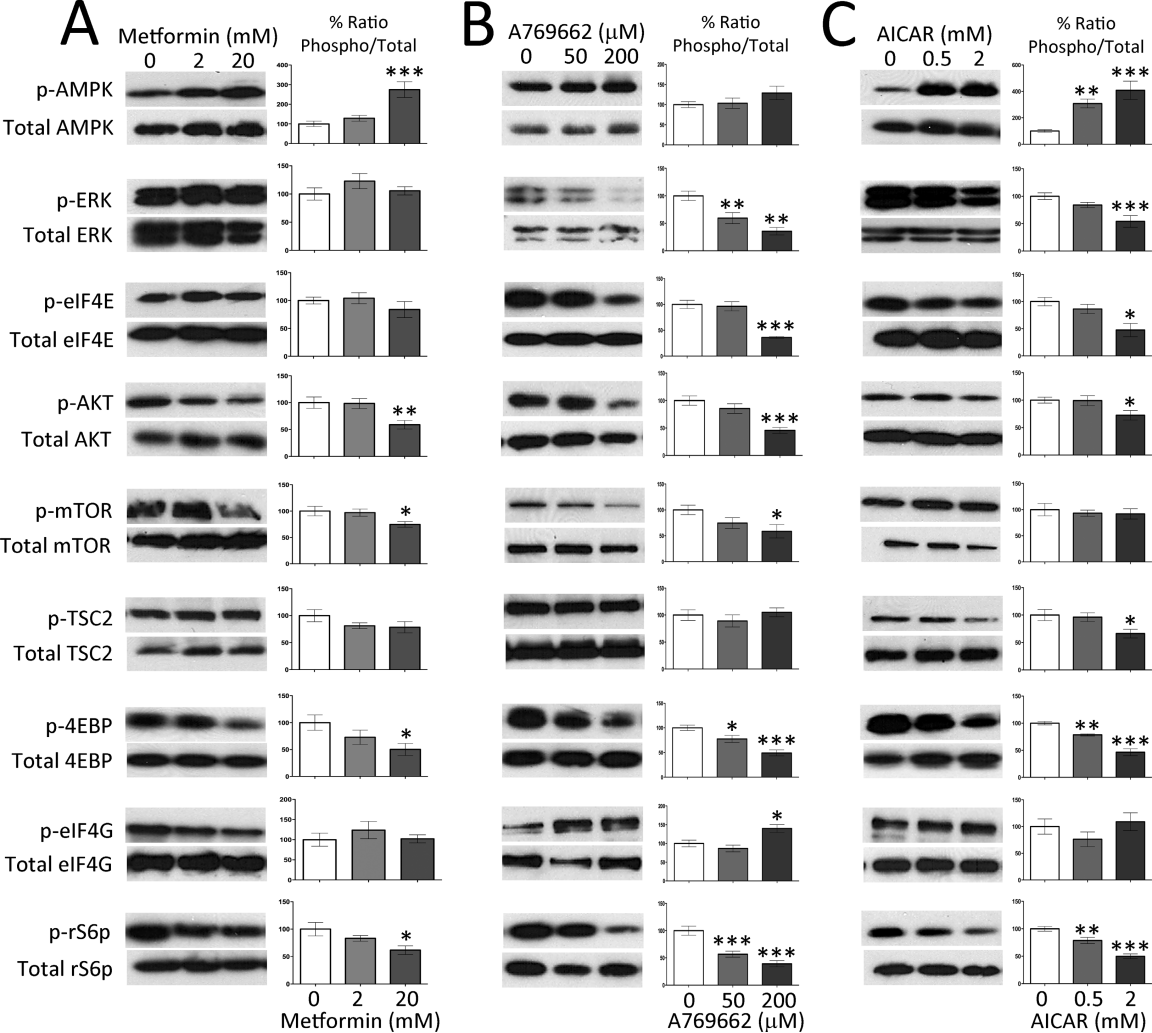
**Treatment with AMPK activators suppresses translation regulation signaling**. **A) **Treatment of mouse sensory neurons cultured in the presence of NGF (50 ng/ml) with metformin (2 and 20 mM) for 1 hour induces a dose-dependent increase in the phosphorylation of AMPK. Metformin treatment abrogates the phosphorylation of mTOR, 4EBP and rS6 in a dose-dependent manner. Metformin does not suppress the ERK-eIF4E pathway. **B) **AMPK allosteric activator A769662 suppresses translation regulation signaling. Treatment of mouse sensory neurons with A769662 (50 and 500 μM) results in a dose dependent suppression of phosphorylation of ERK, eIF4E, mTOR, 4EBP, AKT and rS6. **C) **Treatment of mouse sensory neurons with AICAR (0.5 and 2 mM) for 1 hour results in a dose dependent activation of AMPK. Moreover, AICAR dose-dependently suppresses the phosphorylation of ERK, eIF4E, AKT, TSC2, 4EBP and rS6. N = 6 per group. *p < 0.05, **p < 0.01 and ***p < 0.001.

**Figure 5:**
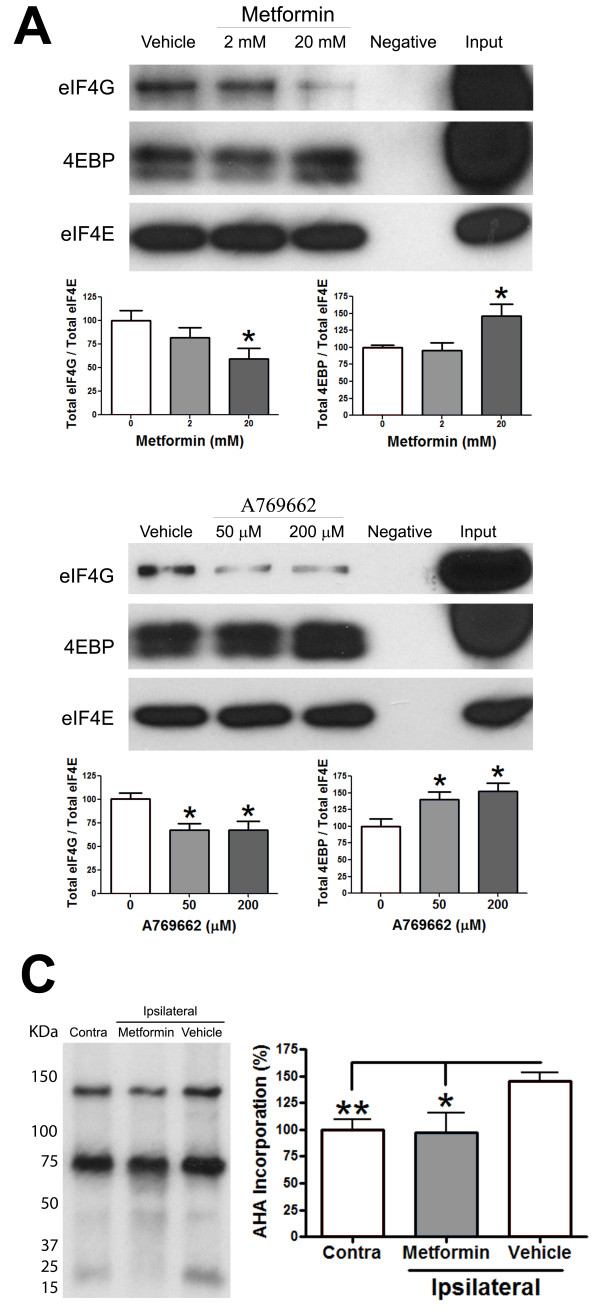
**AMPK activators suppress translation in sensory neurons and the injured PNS**. Treatment of mouse sensory neurons cultured in the presence of NGF (50 ng/ml) with **A) **Metformin (2 and 20 mM) or **B) **A769662 (50 and 500 μM) results in decreased binding of eIF4G and increased 4EBP binding to the m7GTP-cojugated sepharose beads in a dose-dependent manner consistent with a decrease in eIF4F complex formation. **C) **Metformin treatment of rats with SNL restores nascent protein synthesis in injured nerves to levels observed in uninjured nerves. N = 6 per group. *p < 0.05, ** p < 0.01.

We asked if AMPK activators inhibit signaling pathways associated with translation regulation in sensory neurons in the presence of nerve growth factor (NGF). We used mouse TG neurons for these studies because we have previously shown that NGF signaling to ERK and mTOR *in vitro *is identical in TG and DRG neurons from this species [7]. Utilizing TG neurons greatly reduces the number of animals required for these studies. NGF promotes mTOR and ERK signaling pathways in these neurons [7], contributes to neuropathic pain in preclinical models [22] and is associated with neuropathic pain in humans [23]. Mouse TG neurons were treated with metformin (Figure 4A), A769662 (Figure 4B) or AICAR (Figure 4C) for 1 hour. A769662 and AICAR activated AMPK and suppressed activity in the mTOR and ERK pathways whereas metformin activated AMPK and selectively inhibited the mTOR pathway but did not lead to feedback signaling through IRS (e.g. no increase in ERK or AKT phosphorylation [24]). Moreover, A769662 and metformin inhibited eIF4F complex formation (Figure 5A and 5B) in primary cultures of TG neurons treated with NGF. To determine if metformin inhibited dysregulated translation *in vivo*, we excised sciatic nerves from SNL rats treated with vehicle or metformin (200 mg/kg/day for 7 days). A 50% increase in nascently synthesized proteins was observed in the sciatic nerve of SNL rats treated with vehicle, whereas metformin treatment restored nascently synthesized protein levels to those observed in uninjured sciatic nerves (Figure 5C). Hence, AMPK activators suppress translation regulation pathways in sensory neurons and inhibit nascent protein synthesis in the sciatic nerve associated with PNI.

### AMPK activators block hyperexcitability in sensory neurons

**Figure 6:**
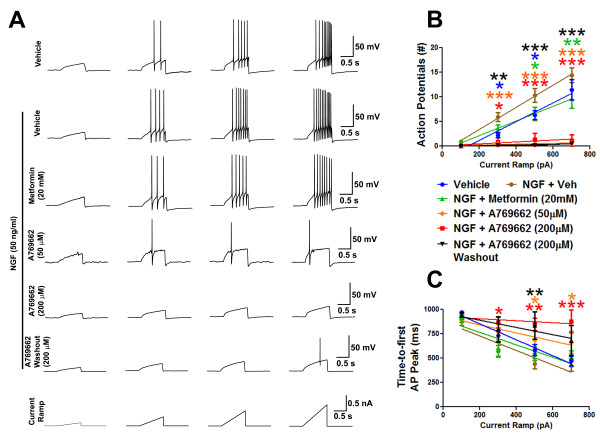
**AMPK activators suppress hyperexcitability of sensory neurons**. **A) **Patch clamp analysis of mouse primary sensory neurons cultured in the presence of NGF (50 ng/ml, n = 13) demonstrate an increase in the **B) **number of ramp currentevoked action potentials vs. vehicle (n = 14) and **C) **reduced latency to first action potential in response to ramp currents. Metformin (1 hr, n = 14) and A769662 (1 hr, n =12) reverse these parameters. A769662 effect persists after washout (n = 8). Colored stars denote significant effects compared to the NGF + Vehicle group. *p < 0.05, **p < 0.01 and ***p < 0.001.

Based on the biochemical findings above, we hypothesized that AMPK activators might attenuate ramp current-evoked sensory neuron excitability. Small diameter (20-30 pF), mouse TG neurons cultured in the presence of NGF displayed increased excitability when stimulated with depolarizing ramp current injections (Figure 6A, B and 6C). Treatment with metformin restored these parameters to levels observed in vehicle-treated cultures (Figure 6A, B and 6C). Treatment with A769662 dose-dependently prolonged latency to first action potential (Figure 6C) and potently reduced the number of action potentials in response to ramp currents (Figure 6A). This effect persisted for at least 1hr after washout (Figure 6) demonstrating that the observed effects are not due to direct channel blockade. AMPK activators did not significantly influence resting membrane potential (vehicle = -60.52 ± 0.74; NGF + vehicle = -60.61 ± 0.70; NGF + metformin = -61.27 ± 1.22; NGF + A769662 (50 μM) = -58.3 ± 1.63; NGF + A769662 (200 μM) = -56.13 ± 2.37) after one hr exposure. As noted in biochemical experiments, A769662 suppressed ERK activation in sensory neurons (Figure 4B) whereas metformin only influenced the mTOR pathway (Figure 4A). This discrepancy in signaling, which likely reflects differential pharmacological upstream mechanisms of these compounds [19,21], may provide insight into the enhanced efficacy of A769662 in reducing sensory neuron excitability in response to ramp currents. Thus, AMPK activators not only suppress translation regulation signaling and eIF4F complex formation but also decrease the excitability of sensory neurons.

## Discussion

The present findings provide several novel insights into the pathology and potential treatment of neuropathic pain. We have demonstrated that PNI induces a major reorganization of translation regulation signaling, translation machinery and RNA-binding proteins in the injured PNS. These changes are directly linked to increased eIF4F complex formation and augmented nascent protein synthesis. We have identified AMPK activation as a novel avenue for the potential treatment of neuropathic pain in humans. Metformin and A769662 both alleviated neuropathic allodynia, inhibited translation regulation pathways associated with PNI and decreased sensory neuronal excitability. No clinical trials have assessed the efficacy of metformin for neuropathic pain. These studies provide a compelling preclinical rationale for the clinical assessment of metformin for neuropathic pain in humans and for the future development of more efficacious AMPK activators for the treatment of chronic pain disorders.

Translation control in the axonal compartment of neurons contributes to development of the peripheral and central nervous systems [25], is involved in axonal regeneration following injury [26,27] and contributes to pain plasticity [7,8]. Likewise, the repertoire of mRNAs localized to the axonal compartment is developmentally regulated and shows plasticity upon injury [26-30]. Interestingly, a recent non-biased approach to mRNA profiling of the axonal compartment of DRG neurons revealed a developmental shift toward localization of mRNAs involved in immune regulation and nociception in the adult DRG axon [28]. In support of this mRNA profile, we have recently demonstrated that NGF and/or IL-6-induced allodynia is dependent on local, axonal translation from existing pools of axonally localized mRNAs [7]. The present findings enhance our understanding of plasticity of translation control after PNI. While changes in mRNA localization have been observed after pre-conditioning peripheral nerve lesions [26], we found that PNI induces profound changes in activity of kinases associated with translation control (e.g. mTOR and ERK), phosphorylation of their downstream targets and in overall levels of proteins involved in RNA processing and transport (e.g. Mov10, FMRP and rck/p54). This reorganization results in increased eIF4F complex formation and nascent protein synthesis in the injured sciatic nerve. Moreover, these changes are directly correlated to normalization of neuropathic allodynia as metformin reversed changes in protein synthesis in injured sciatic nerves and resolved PNI-induced allodynia.

Protein synthesis is an energy intensive process and, for this reason, an intricate system to control energy consumption has evolved in the form of the ubiquitous kinase, AMPK [11]. Activated AMPK blocks protein synthesis by inhibiting components of the mTOR and ERK signaling pathways [11]. Concurrent inhibition of multiple signaling pathways inherently prevents signaling crosstalk, which is commonly observed with the inhibition of a single kinase involved in these signaling cascades (e.g. inhibition of mTORC1 with rapamycin [24]). Receptor tyrosine kinases are associated with IRS, which mediates activation of the mTOR and ERK pathways. Inhibition of mTORC1 with rapamycin removes negative feedback onto IRS, meditated by phosphorylation of IRS (S1101) by rS6K [31]. Thus, inhibiting mTORC1 and, in turn, rS6K, with rapamycin releases disinhibition of IRS signaling resulting in activation of ERK and mTORC2/AKT pathways [11]. ERK activation in the PNS is a well known mechanism for increasing the excitability of nociceptors [32]. For these reasons we focused on AMPK as a therapeutic target for neuropathic pain as AMPK suppresses IRS signaling by phosphorylation on Serine 794 [17]. Activation of AMPK with distinct pharmacological tools failed to promote ERK or AKT activation and, in the case of A769662 and AICAR, led to inhibition of these kinases. These findings highlight advantages of targeting AMPK for the treatment of neuropathic pain.

We have also demonstrated that activation of AMPK in mouse sensory neurons leads to decreased excitability. We used ramp current-evoked spiking to assess the influence of AMPK activation on sensory neuron excitability. Our findings are consistent with a potential modulation of the voltage-gated sodium channel Nav1.7 by AMPK activators. Human genetic studies clearly demonstrate an important role for Nav1.7 in inherited pain conditions and a growing body of evidence suggests an important role for Nav1.7 in acquired chronic pain states [33]. In humans, Nav1.7 expression is increased in painful neuromas [34] and dental pulp [35,36]. Moreover, inhibition of Nav1.7 decreases sensory neuron excitability [37,38]. Genetic deletion of Nav1.7 in mice leads to marked decreases in acute and inflammatory pain [39]. Finally, pharmacological inhibition of Nav1.7 with several distinct classes of inhibitors leads to a reduction in allodynia in preclinical neuropathic pain models [40-43]. Hence, human clinical findings and pharmacological inhibition of Nav1.7 creates a compelling rationale for targeting Nav1.7 in neuropathic pain disorders. The present findings indicate that the AMPK signaling axis regulates sensory neuronal activity by decreasing action potential firing induced by ramp current injection and increasing the latency to the first action potential, both of which are consistent with modulation of Nav1.7 [44]. We hypothesize that this AMPK-mediated modulation of sensory neuron excitability may be linked to inhibition of ERK because ERK phosphorylates Nav1.7 altering channel gating properties toward a hyperexcitable state and leads to decreased neuronal hyperexcitability [44]. While further work will be needed to directly test the effect of AMPK activators on ERK-mediated Nav1.7 phosphorylation and Nav1.7 current kinetics, the present findings demonstrate a role for AMPK modulation in sensory neuronal excitability.

The results presented here are consistent with a peripheral action for AMPK activators in the alleviation of SNI- and SNL-induced allodynia; however, we cannot exclude a potential central mechanism of action. Several recent studies have demonstrated an important role for dorsal horn mTOR in preclinical pain models [9,45-49] and AMPK activators influence the mTOR pathway in central neurons [50,51]. Moreover, metformin crosses the blood brain barrier [52]. We favor a peripheral mechanism of action for several reasons. AMPK activators had a clear effect on mTOR and, in some cases, ERK signaling, in cultured sensory neurons. These compounds also negatively influenced the excitability of these neurons, consistent with the alleviation of neuropathic pain. Moreover, in vivo treatment led to a reversal of PNI-induced enhanced nascent protein synthesis, consistent with a direct action of AMPK activators on the injured PNS. Finally, inhibition of translation regulation signaling (e.g. with mTORC1 inhibitors) in the CNS is thought to play a critical role in the initiation but not maintenance of plasticity [53]. To this end, we have recently shown that mTOR inhibiton in the dorsal horn is incapable of reversing an established preclinical pain state [47] and, in the setting of neuropathic pain, other investigators have concluded that even centrally applied mTOR inhibitors act via inhibition of DRG neuron excitability [4]. Nevertheless, we cannot rule out a potential CNS site of action for AMPK activators and, due to a key role of translation regulation in learning and memory [3] we also cannot rule out a possible effect of AMPK activators on these processes. Additional pharmacokinetic/pharmacodynamic studies will be required to resolve this question with certainty.

The present findings have important implications for AMPK-based drug discovery for the treatment of pain. Metformin activates AMPK via LKB1 stimulation [19] and inhibition of AMP deaminase [18]. AICAR results in 5-aminoimidazole-4-carboxamide ribonucleoside accumulation in cells mimicking AMP binding to AMPK [54]. On the other hand, A769662 is a direct, positive allosteric modulator of AMPK [21] which requires the β1 subunit of the kinase heteromer [55]. Unlike metformin and AICAR which activate AMPK by inducing the phosphorylation T172 on the alpha subunit, A769662 induced AMPK activation does not require this post-translational modification [56]. While both metformin and A769662 led to a full reversal of neuropathic allodynia, A769662 was more potent *in vivo *and had a more profound effect on sensory neuron excitability with a near complete blockade of ramp current evoked spiking. Moreover, A769662 (and AICAR) led to robust ERK inhibition in sensory neurons in culture whereas metformin had no impact on ERK activity. While further experimentation will be needed to gain insight into the exact mechanisms through which different modes of AMPK activation achieve differential signaling endpoints, these results point to a potential pharmacological advantage for positive allosteric modulators of AMPK for the treatment of chronic pain. Furthermore, the efficacy of A769662 in mouse models of neuropathic pain suggests that targeting the β1-subunit of AMPK may be a viable drug development target for the pain pathway.

In conclusion, we have demonstrated a novel pathway for the potential treatment of neuropathic pain, AMPK activation. Pharmacological AMPK activation negatively regulates aberrant translation control after PNI, resolves neuropathic allodynia and decreases sensory neuron excitability. Due to the clinical availability and safety of metformin, these preclinical findings have the potential to lead to rapid translation into the clinic.

## Methods

### Surgery and behavioral testing

Male ICR mice (Harlan, 20-25 g) and male Sprague Dawley rats (Harlan, 250-300 g) were used. All animal procedures were approved by the Institutional Animal Care and Use Committee of The University of Arizona and were in accordance with International Association for the Study of Pain guidelines. Prior to surgery all animals were assessed for mechanical withdrawal thresholds [57]. Spared nerve injury (SNI) was performed on the mice as described previously [58]. Spinal nerve ligation (SNL) was done on rats by tight ligation of the L5 and L6 spinal nerves as described by Kim and Chung [59]. Sham control animals underwent the same surgery and handling as the experimental animals but without the SNL or SNI. All animals were allowed to recover for 14 days and all testing commenced day 14 day post-surgery (except as noted in the text when testing was done 7 weeks post SNI). Following nerve injury, only animals that developed paw withdrawal thresholds less than 1 g for SNI and less than 4.7 g for SNL by day 14 post-surgery were used. Animals were placed in acrylic boxes with wire mesh floors and allowed to habituate for 1 hr. Pre-drug mechanical thresholds were recorded and the animals received intraperitoneal injections of vehicle, metformin (200 mg/kg) [20] or A7969662 (30 mg/kg) [21]. Calibrated von Frey filaments (Stoelting, Wood Dale, IL) were used for mechanical stimulation of the plantar surface of the left hindpaw and withdrawal thresholds were calculated using the up-down method [57]. For Western blotting, eIF4F complex formation and nascent protein synthesis studies, tissues were harvested 17 days post SNL or SNI unless drug treatments were initiated on day 14, in which case they were harvested on day 21.

### Primary neuronal cultures

Mouse trigeminal ganglia (TG) were excised aseptically and placed in Hank's Buffered Salt Solution (HBSS, Invitrogen) on ice. The ganglia were dissociated enzymatically with collagenase A (1 mg/ml, 25 min, Roche) and collagenase D (1 mg/ml, Roche) with papain (30 U/ml, Roche) for 20 min at 37°C. To eliminate debris 70 μm (BD) cell strainers were used. The dissociated cells were resuspended in DMEM/F12 (Invitrogen) containing 1× pen-strep (Invitrogen), 1× GlutaMax, 3 μg/ml 5-FDU (Sigma), 7 μg/ml uridine (Sigma), 50 ng/ml NGF (Millipore) and 10% fetal bovine serum (Hyclone). The cells were plated in 6-well plates (BD Falcon) and incubated at 37°C in a humidified 95% air/5%CO2 incubator. On day 5 the cells were washed in DMEM/F12 media for 30 mins followed by treatment.

### Western blotting

Protein was extracted from the cells and tissue in lysis buffer (50 mM Tris HCl, 1% Triton X-100, 150 mM NaCl, and 1 mM EDTA at pH 7.4) containing protease and phosphatase inhibitor mixtures (Sigma) with an ultrasonicator on ice, and cleared of cellular debris and nuclei by centrifugation at 14,000 RCF for 15 min at 4°C. Fifteen micrograms of protein per well were loaded and separated by standard 7.5% or 10% SDS-PAGE. Proteins were transferred to Immobilon-P membranes (Millipore) and then blocked with 5% dry milk for 3 h at room temperature. The blots were incubated with primary antibody overnight at 4°C and detected the following day with donkey anti-rabbit antibody conjugated to horseradish peroxidase (Jackson Immunoresearch). Signal was detected by ECL on chemiluminescent films. Each phosphoprotein was normalized to the expression of the corresponding total protein which in turn were normalized to GAPDH and βIII tubulin on the same membrane. Membranes were stripped prior to antibody incubation for normalization. Densitometric analyses were performed with Image J software (NIH).

### Enzyme Linked Immunosorbent Assay (ELISA)

ELISAs for NGF (Promega) and IL-6 (Thermo) were performed on the sciatic nerves form rats with SNL according to the manufacturer's instruction. The optical density was read by a plate reader (Multiskan Ascent, Themo) using a 450 nm filter. Ascent Software (Themo) was used for the data analysis and cubic spline curve fit was chosen for that purpose.

### 5' mRNA cap complex analysis

After the protein extraction, 50 μg protein was incubated with 7- methyl GTP Sepharose 4B beads (GE Healthcare) in the presence of 100 μM GTP for 2 h at 4°C. Unconjugated sepharose 4B beads were used for the negative controls. The beads were then pelleted and washed twice with lysis buffer. After a final centrifugation the pellet was suspended in 1× Laemmli Sample Buffer containing 5% v/v β-mercaptoethanol and eIF4E, eIF4G eIF4A and 4EBP bound to the precipitated beads was analyzed by Western blotting.

### Immunohistochemistry

Slide-mounted sections were fixed in ice-cold 3.7% paraformaldehyde in 1× PBS for 1 h and then washed 3 times for 5 min in PBS. Slides were transferred to a solution containing 0.1 M sodium citrate and 0.05% Tween 20 and then microwaved on high power for 3 min in a 900 W microwave oven for antigen retrieval. After a 30 min cooling period, slides were again transferred to 1× PBS, washed 3 times for 5 min, and then permeabilized in 1× PBS, containing 0.05% Triton X-100. Slides were then blocked for at least 1 h in 1× PBS, containing 10% normal goat serum, before the addition of anti-phospho-mTOR antibody overnight at 4°C. The antiperipherin and N52 antibodies were applied together. Immunoreactivity was visualized after subsequent incubation with goat anti-rabbit and goat anti-mouse Alexa-Fluor antibody for 1 h at room temperature. All immunohistochemistry (IHC) images are representative of samples taken from three animals. Confocal IHC micrographs were acquired on Ziess LSM 510 META NLO upright microscope using a 40×, 1.3 numerical aperture oil immersion objective.

### Nascent protein synthesis in sciatic nerves

Ipsilateral and contralateral sciatic nerves from SNL rats or ipsilateral sciatic nerves from sham rats were excised at a length of 2 cm. The nerves were then cut to a length of 1 cm and incubated in DMEM/F12 supplemented with 50 μM of Azidohomoalanine (AHA). After 2 hours of incubation at 37°C in a humidified 95% air/5%CO2 incubator, protein was extracted from the nerves by ultrasonication in RIPA buffer. AHA incorporating proteins were labeled with biotin using Click-iT Biotin Protein Analysis Detection Kit (Invitrogen). The samples were analyzed using western blotting with the biotin labeled proteins detected by avidin-HRP chemiluminescence.

### Patch clamp electrophysiology

Whole cell patch-clamp experiments were performed on isolated mouse TG using a MultiClamp 700B (Axon Instruments) patch-clamp amplifier and pClamp 10 acquisition software (Axon Instruments). Recordings were sampled at 5 kHz and filtered at 1 kHz (Digidata 1322A, Axon Instruments). Pipettes (OD: 1.5 mm, ID: 0.86 mm, Sutter Instrument) were pulled using a P-97 puller (Sutter Instrument) and heat polished to 2.5-4 MΩ resistance using a microforge (MF-83, Narishige). Pipette offsets were zeroed automatically before seal formation and liquid junction potentials were not corrected. Pipette capacitance neutralization and bridge balance were adjusted automatically in current-clamp mode. All recordings were performed at room temperature. Data were analyzed using Clampfit 10 (Molecular Devices) and Origin 8 (OriginLab). Pipette solution contained (in mM) 140 KCl, 11 EGTA, 2 MgCl2, 10 NaCl, 10 HEPES, 1CaCl2 pH 7.3 (adjusted with N-methyl glucamine), and was 320 mosM. External solution contained (in mM) 135 NaCl, 2 CaCl2, 1 MgCl2, 5 KCl, 10 Glucose and 10 HEPES, pH 7.4 (adjusted with N-methyl glucamine), and was 320 mosM. The cells were treated with relevant drug for 1 hour before patch clamp studies were carried out. Ramp stimulus protocols for Nav1.7 were performed as described previously [44] as these protocols have been shown to preferentially elicit function from Nav1.7 channels [60]. The viability of the neurons was verified by eliciting currents in response to a voltage step protocol before and after each current ramp protocol was carried out. Al recordings were made on mouse TG neurons from ~20 gram mice. Capacitance of all cells analyzed was between 20-30pF.

### Drugs and primary antibodies

U-0126 and U0124 were from Tocris; metformin was from LKT Laboratories; mouse 2.5S NGF was from Millipore; The following rabbit polyclonal antibodies were obtained from Cell Signaling: p-ERK (Thr202/Tyr204, cat# 4377), total ERK, p-eIF4E (Ser209, cat# 9741), total eIF4E, p-mTOR (Ser2448, cat# 2971), total mTOR, p-4EBP(Thr37/46, cat # 9459), total 4EBP, p-eIF4G (Ser1108, cat# 2441), total eIF4G, p-AKT (Ser473, cat# 4058), total AKT, GAPDH and eIF4A. Mov10 was from Bethyl Labs and rck/p54 was from MBL international. A769662 was from LC Laboratories.

### Statistical Analysis and Data Presentation

Data are shown as means and the standard error of the mean (± SEM) of eight independent cell culture wells, 6 tissue samples (for *in vivo *Western blotting, eIF4F complex formation and nascent protein synthesis) or 6 animals (for behavioral studies). Graph plotting and statistical analysis used Graphpad Prism Version 5.03 (Graph Pad Software, Inc. San Diego, CA, USA). Statistical evaluation was performed by one- or two-way analysis of variance (ANOVA), followed by appropriate post-hoc tests. The a priori level of significance at 95% confidence level was considered at p < 0.05.

## Competing interests

The authors declare that they have no competing interests.

## Authors' contributions

OKM, AK, NS, GD and TJP conceived of the study and designed experiments, OKM, MNA, DVT, RS, JY, AL, JJ, KAP and TL performed experiments. OKM, MNA, GD and TJP analyzed data. OKM, NS, GD and TJP wrote the manuscript. All authors read and approved the final manuscript.
